# Osteogenic Response of Dental Pulp Stem Cells (DPSCs) to an Alginate/Chitosan/Cannabidiol Hydrogel

**DOI:** 10.3390/gels12090843

**Published:** 2026-09-15

**Authors:** Hernan Santiago Garzon, Lina Suárez, Daniel Suárez

**Affiliations:** 1 Programa Doctorado en Ingeniería, Facultad de Ingeniería, Pontificia Universidad Javeriana, Bogotá 110231, Colombia; 2 Departamento de Ciencias Básicas y Medicina Oral, Facultad de Odontología, Universidad Nacional de Colombia, Bogotá 111321, Colombia; 3 Grupo de Investigación en Ciencias Odontológicas, Programa de Especialización en Periodoncia, Institución Universitaria Colegios de Colombia, Colegio Odontológico Colombiano, Bogotá 111071, Colombia; 4 Centro de Investigaciones Odontológicas, Departamento del Sistema Periodontal, Facultad de Odontología, Pontificia Universidad Javeriana, Bogotá 110231, Colombia; 5 Departamento de Ingeniería Industrial, Facultad de Ingeniería, Pontificia Universidad Javeriana, Bogotá 110231, Colombia

**Keywords:** bone tissue engineering, dental pulp stem cells, alginate/chitosan hydrogel, cannabidiol, osteoprotegerin, RANKL

## Abstract

This study evaluated the osteogenic differentiation and paracrine response of dental pulp stem cells (DPSCs) cultured within a 3D-printed alginate/chitosan/cannabidiol (CBD) hydrogel. DPSCs from a single donor (passage 6; n = 3 technical replicates per condition) were cultured as monolayers or on hydrogel constructs (CBD 0–12 mg/mL) in basal or osteogenic medium for 7, 14, and 21 days. Alizarin Red S staining confirmed retention of osteogenic competence. Supernatant osteoprotegerin (OPG) and RANKL were quantified by Luminex immunoassay. Monolayer cultures produced up to 7867.5 ± 676.8 pg/mL OPG, whereas hydrogel cultures yielded 9.7–69.3 pg/mL (98–99.8% reduction; *p* < 0.001). RANKL remained stable at 9–11 pg/mL across all conditions; neither analyte varied with CBD concentration. The OPG/RANKL ratio collapsed from 215–858 to 1.0–6.3 in hydrogel groups. Since RANKL—co-secreted by the same cells—was unaffected, a generalized cellular or secretory deficit is unlikely to explain the selective OPG decrease. The data are consistent with charge-selective retention of cationic OPG (pI ≈ 8.5–9.0) within the anionic alginate network, although this mechanism remains hypothetical pending direct adsorption and ζ-potential verification. These findings indicate that scaffold fixed charge constitutes an active variable in the apparent secretome of three-dimensional cultures.

## 1. Introduction

Bone tissue regeneration is challenging, especially for critical defects that exceed the capacity of natural repair. Scaffolds are vital in such cases, as the body cannot always restore extensive bone loss resulting from trauma, surgery, infections, or degenerative diseases [1]. Bone defects in the maxillofacial and oral cavity commonly result from trauma and conditions such as periodontitis, which lead to significant bone loss. Rising maxillofacial injuries, particularly mandibular fractures, are frequently observed among women aged 21–30 due to cycling and motorcycle accidents [2,3]. In 2021, over one billion individuals worldwide had periodontitis, with a 12.50% global prevalence and nearly 90 million new cases [4]. Treatments for oral cancer often require extensive bone resections, resulting in critical defects [5].

Recent advances in bone tissue engineering focus on 3D-printed scaffolds that mimic tissue’s biological, mechanical, and chemical features. These include implants and scaffolds loaded with drugs, proteins, growth factors, and biomolecules such as BMPs, as well as cell therapy, all of which aim to promote bone growth and function [6]. Hydrogels, formed by crosslinking hydrophilic polymers, are used for delivering bioactive compounds. They support tissue engineering, drug delivery, and healing by maintaining nutrient levels and promoting angiogenesis, achieved via foaming, lyophilization, electrospinning, and 3D printing using materials such as chitosan, gelatin, hyaluronic acid, PLA, PVA, and PEG [7].

Hydrogels are a promising option in bone tissue engineering due to their hydrophilic, biocompatible 3D polymer networks and customizable porous structures. These features help cell infiltration and tissue integration [8]. Key properties such as surface, adhesion, mechanics, porosity, and degradation influence cell migration, growth, and differentiation, thereby affecting bone regeneration [9,10]. Hydrogels also trigger regenerative biological processes and can mimic the natural extracellular matrix of bone. For example, dynamic GelMA/DNA systems support bone organoid formation and in vivo regeneration. They are also essential for immune responses, particularly by guiding macrophage polarization toward the M2 phenotype, fostering regeneration, and accelerating healing [11].

Extensive evidence indicates that alginate and chitosan can form hydrogels via electrostatic interactions, yielding stable, tunable networks. This polymer blend offers high biocompatibility, controlled biodegradation, in situ gelation, and promotes cell adhesion and growth [12,13,14]. Its porous, hydrophilic structure enhances the diffusion of nutrients and oxygen, supporting angiogenesis and cell migration. Both polymers, alone and in combination, have antimicrobial, anti-inflammatory, wound-healing, and hemostatic properties, which are vital for bone defects at risk of infection. Therefore, the development of alginate and chitosan hydrogels is promising for bone regeneration [15,16,17].

Beyond their structural role, each component of this ternary system contributes a distinct biological function relevant to bone regeneration. Alginate, through its guluronate blocks, chelates Ca^2+^ to form egg-box junctions that generate a densely anionic network; this fixed negative charge governs how soluble mediators partition between the matrix and the surrounding medium, a property already exploited for sustained delivery of cationic growth factors such as BMP-2 [18]. Chitosan contributes protonated amino groups that complex with alginate carboxylates, thereby improving mechanical stability and cell adhesion, and providing intrinsic antibacterial and pro-osteogenic activity [12,13,14,15,16,17]. Cannabidiol (CBD) provides the biological signal: it induces osteoblast differentiation through the p38 MAPK pathway [19], accelerates callus mineralization in vivo [20], enhances viability and osteogenic gene expression in human skeletal progenitor cells [21], and restores osteo/odontogenic differentiation of DPSCs suppressed by TNF-α or LPS [22,23]. Compared with gelatin/GelMA hydrogels (photo-cross-linked, low fixed charge), collagen matrices (mechanically weak, costly), or synthetic polyesters (harsh processing, unsuitable for cell-laden extrusion), alginate/chitosan gels ionically at physiological temperature, is extrusion-printable, and carries a marked charge asymmetry that may itself shape the pericellular protein environment [24,25,26,27].

Natural medicinal compounds are popular for reducing the side effects of synthetic medicine and treating bone disorders. Used holistically, about 40% of US adults use them. Their diverse activity, resistance-free nature, and simple therapy set them apart from synthetics. Overcoming bone tissue engineering challenges involves integrating these compounds with modern technologies, such as incorporating CBD into hydrogels. CBD’s anti-inflammatory, immunomodulatory, and regenerative properties make it a promising development [19,28,29]. Recent research shows CBD helps normalize fracture pain, boost periosteal progenitors, accelerate callus mineralization, and increase bone density in mice [20]. CBD also enhances the viability and growth of human skeletal progenitors, regulates osteocalcin levels, and prevents osteoporosis in ovariectomized mice [21].

MSCs (Mesenchymal Stem Cells) are clonally capable of self-renewal and differentiation into various lineages. They migrate to sites of inflammation, releasing immunosuppressive and anti-inflammatory molecules. Their availability and ease of expansion make them suitable for treating orthopedic injuries, cardiovascular diseases, neurodegenerative disorders, autoimmune diseases, and liver conditions [30]. MSCs are isolated from tissues like bone marrow, blood, and dental tissue, especially dental pulp (DPSC). Known as the ‘gold standard,’ they proliferate highly and can differentiate into osteoblasts, muscle cells, adipocytes, chondrocytes, and corneal epithelial cells [31].

hDPSCs can differentiate into cell types such as odontoblasts, osteoblasts, chondrocytes, cardiomyocytes, neurons, and adipocytes by modulating the levels of growth factors, transcription factors, ECM proteins, and receptor molecules. They mainly originate from cranial neural crest cells, as indicated by markers such as GFAP, HNK-1, nestin, P75, and S-100, which are characteristic of neural crest stem cells. Thus, DPSCs can become neurons, cardiomyocytes, adipocytes, and insulin-secreting beta cells [30].

The osteoprotegerin (OPG)/RANKL axis provides the functional read-out used in this study and is therefore introduced here. Osteoprotegerin is a soluble decoy receptor of the tumor necrosis factor receptor superfamily, secreted mainly by osteoblast-lineage and stromal cells. It binds the receptor activator of nuclear factor-κB ligand (RANKL) and blocks its interaction with RANK on osteoclast precursors, thereby preventing osteoclast differentiation, activation, and survival [32,33]. RANKL, in contrast, is the primary cytokine driving osteoclast formation. The OPG/RANKL ratio expresses the balance between bone-protective and bone-resorptive signaling: a high ratio inhibits osteoclastogenesis and favors bone preservation, whereas a low ratio promotes osteoclast formation and resorption [33]. DPSCs are characterized by a markedly OPG-dominant secretory profile, producing OPG at more than twice the level of alveolar bone cells while secreting roughly twenty-fold less RANKL, which yields a low RANKL/OPG ratio and inhibits osteoclastogenesis by deactivating PI3K-AKT signaling in myeloid cells [34].

Although alginate/chitosan scaffolds, CBD-induced osteoinduction, and the high regenerative potential of DPSCs are well documented, how these three components interact within a single macromolecular network remains poorly understood. Combining the flexible structure of natural polysaccharide hydrogels with the therapeutic effects of phytocannabinoids could create a highly instructive 3D microenvironment that actively influences stem cell fate and local signaling. Clarifying how these three components interact within a single network is therefore essential for advancing cell-based therapies for maxillofacial bone tissue engineering. The objective was to evaluate the osteogenic differentiation capacity of dental pulp stem cells (DPSCs) in contact with the alginate/chitosan/CBD hydrogel.

## 2. Results and Discussion

### 2.1. Determination of the Osteogenic Differentiation of DPSC in Conventional and 3D Cultures

**Figure 1:**
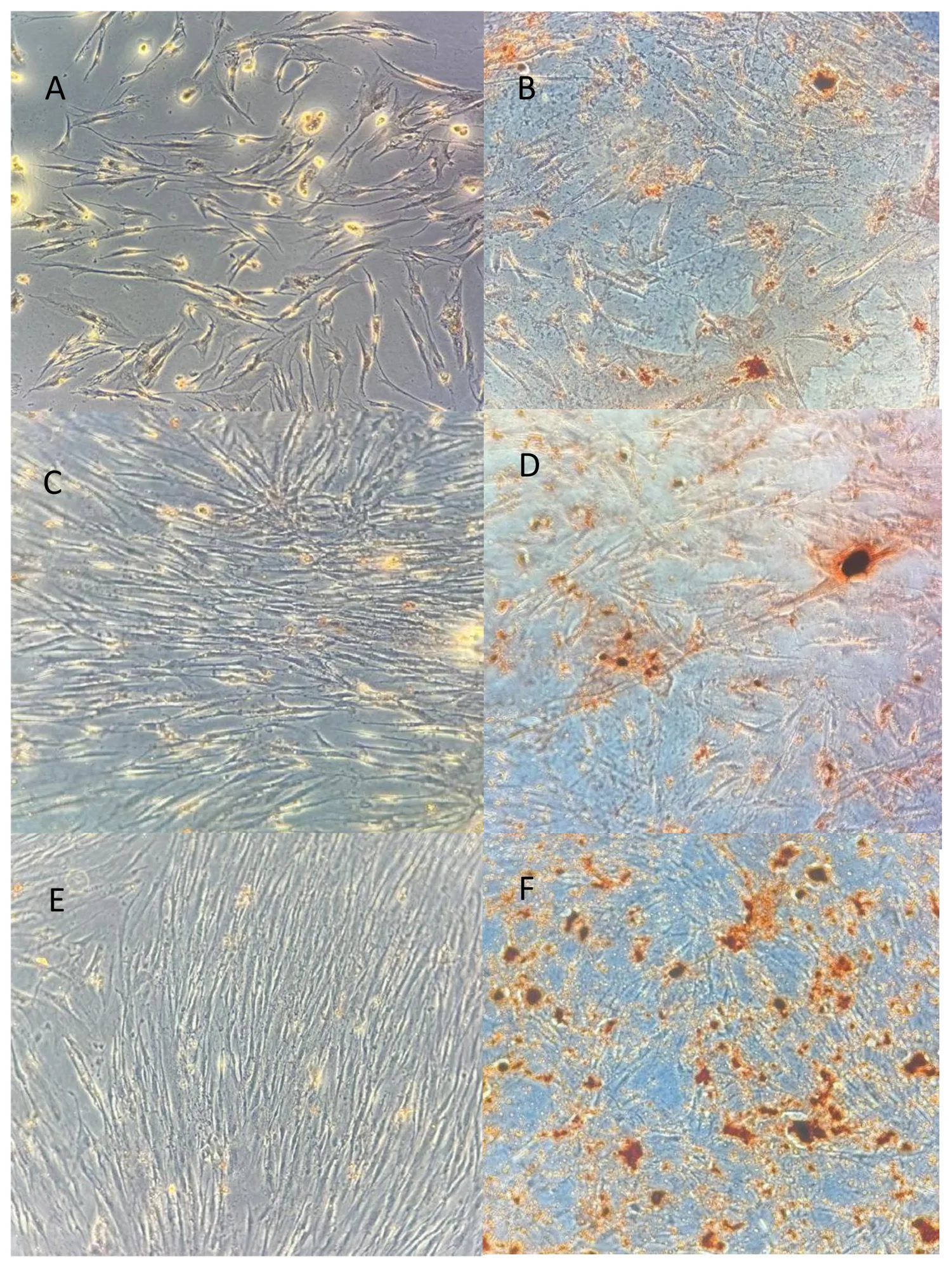
Staining of DPSC cultures with Alizarin Red. In (**A**), the negative control for staining, and in (**B**), the cells were induced to osteodifferentiation and stained with Alizarin Red after 7 days. In (**C**), the negative control for staining, and in (**D**), the cells were induced to osteodifferentiation and stained with Alizarin Red after 14 days. In (**E**), the negative control for staining, and in (**F**), the cells were induced to osteodifferentiation and stained with Alizarin Red after 21 days.

**Figure 2:**
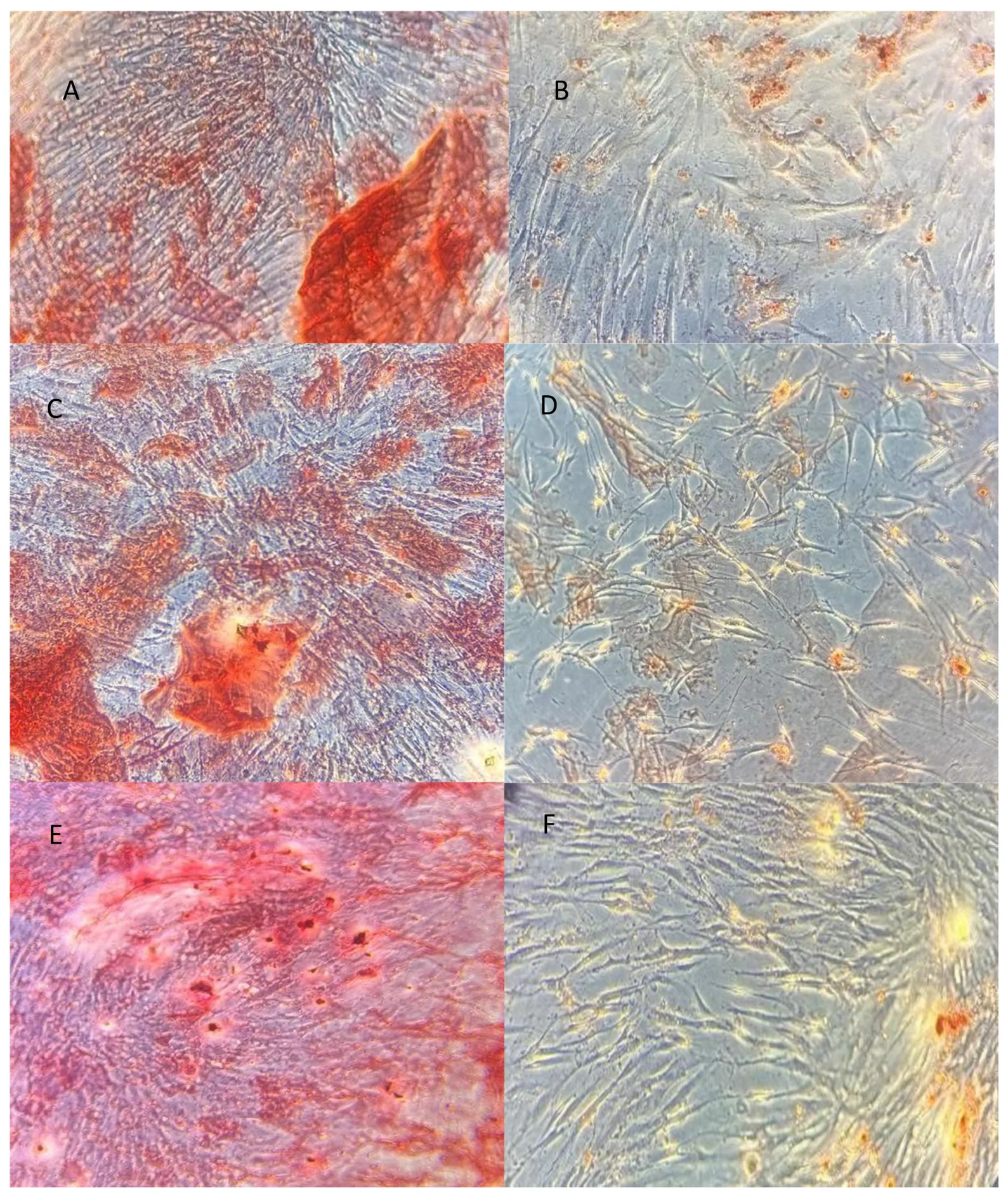
Alizarin Red staining was performed on hydrogels and wells. (**A**) shows a stained hydrogel segment, while (**B**) displays the differentiated cells remaining on the well surface after hydrogel detachment at 7 days. Similarly, (**C**) shows a stained hydrogel segment, and (**D**) shows the remaining differentiated cells at 14 days. On 21 days, (**E**) presents a stained hydrogel segment, and (**F**) shows the differentiated cells on the well surface after detachment. Alizarin Red-positive material is observed at all time points. In panels (**A**,**C**,**E**) the signal arises from both the Ca^2+^-cross-linked matrix and the associated cells; the mineral-specific read-out is restricted to panels (**B**,**D**,**F**), which show the cell layer remaining on the polystyrene surface after hydrogel detachment. All images correspond to the control hydrogel (0 mg/mL CBD).

After each evaluation period, cells were stained using Alizarin Red solution. Figure 1 shows the stained samples at each time point, including their negative controls for osteodifferentiation. In wells with hydrogels, the stain is visible both in the hydrogel and within the proliferating cells, making it difficult to retrieve the hydrogels for optical microscopy. For cells still attached to the well surface, positive staining suggests mineral deposition (Figure 2).

This staining interpretation is limited by two constraints. First, the matrix is ionotropically cross-linked with Ca^2+^, and Alizarin Red S chelates calcium regardless of its biological source; thus, the signal in the gel cannot be attributed solely to cell-derived hydroxyapatite. Since a cell-free hydrogel series spanning the CBD range was not stained in parallel, matrix-derived background cannot be ruled out, and the intragel signal is not definitive evidence of mineral deposition. The mineral-specific readout is confined to the cell layer remaining on the polystyrene surface after hydrogel removal (Figure 2B,D,F), a region free of Ca^2+^-cross-linked polymer, where non-induced cultures kept in basal medium were consistently negative (Figure 1A,C,E). Second, Alizarin Red S stains intragel mineral inconsistently in hydrogel systems, and calcein has been suggested as a more reliable probe [35]. Quantitative assessment would require cell-free hydrogel controls, dye extraction with cetylpyridinium chloride for spectrophotometry [36], and normalization of alkaline phosphatase activity to DNA content. Consequently, staining is regarded as qualitative evidence of osteogenic competence in contact with the hydrogel, not as a measure of mineralization.

### 2.2. OPG/RANKL Production

**Figure 3:**
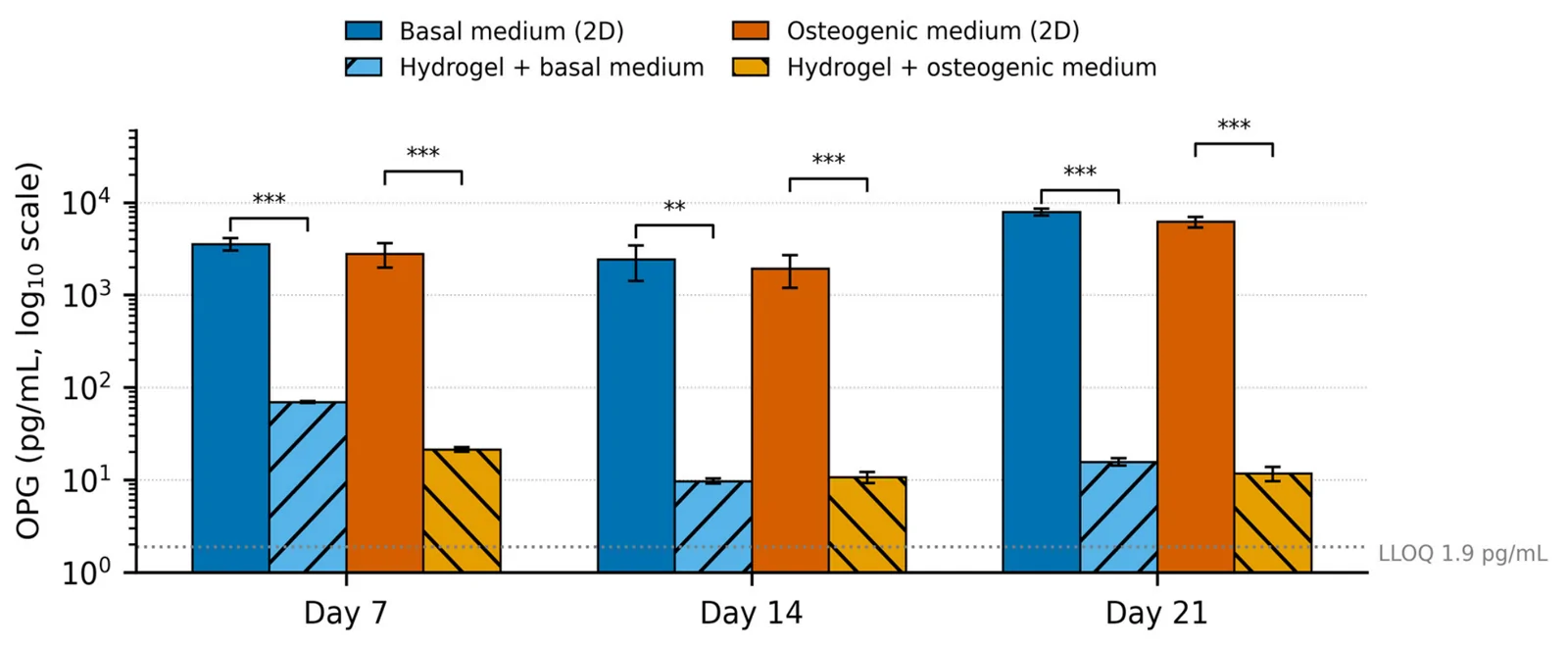
OPG production (pg/mL, mean ± SD) by DPSCs at days 7, 14, and 21 under base medium (BM), hydrogel + BM, osteogenic medium (OM), and hydrogel + OM conditions. Significance brackets: *** *p* < 0.001, ** *p* = 0.005.

A comparison of OPG production by DPSCs showed significant differences between traditional culture conditions and three-dimensional hydrogel setups at all tested time points (Figure 3). On day 7, DPSCs cultured in base medium without hydrogel produced 3558.0 ± 556.2 pg/mL of OPG. When exposed to the control hydrogel (0 mg/mL CBD), this value dropped to 69.3 ± 1.5 pg/mL, a 98% decrease (t = 10.50, *p* < 0.001). In osteogenic medium, OPG levels fell from 2799.8 ± 836.0 pg/mL to 21.3 ± 1.2 pg/mL with hydrogel exposure, indicating a 99% reduction (t = 5.56, *p* < 0.001). This strong suppression persisted at day 14, with control medium cultures showing 2424.2 ± 1014.9 pg/mL compared to 9.7 ± 0.6 pg/mL in hydrogel-treated cells (a 99.6% reduction, t = 3.98, *p* = 0.005), and osteogenic medium cultures presenting 1939.2 ± 746.6 pg/mL versus 10.7 ± 1.5 pg/mL (a 99.4% reduction, t = 4.32, *p* < 0.01; ANOVA F = 11.51, *p* < 0.001). By day 21, the suppression was at its peak, with control medium levels of 7867.5 ± 676.8 pg/mL decreasing to 15.7 ± 1.5 pg/mL (a 99.8% reduction, t = 19.41, *p* < 0.001), and osteogenic medium levels dropping from 6195.0 ± 811.9 pg/mL to 11.7 ± 2.1 pg/mL (also a 99.8% reduction, t = 12.74, *p* < 0.001; ANOVA F = 171.59, *p* < 0.001).

**Figure 4:**
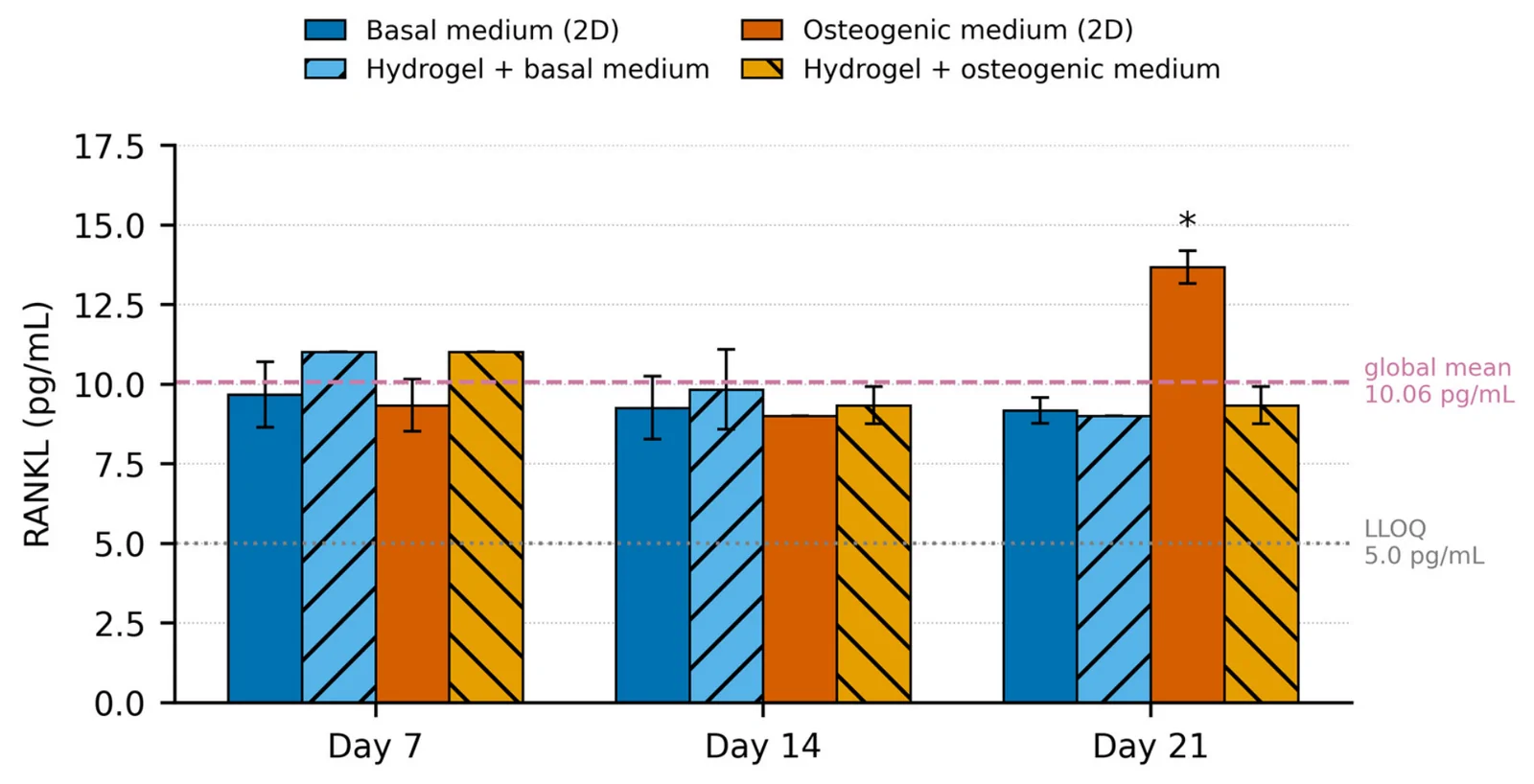
RANKL production (pg/mL, mean ± SD) by DPSCs at days 7, 14, and 21 across all experimental conditions. The red dashed line indicates the global mean (10.06 ± 1.66 pg/mL). The asterisk marks the single statistically significant difference, the osteogenic monolayer group at day 21, whose magnitude is negligible against the 100- to 1000-fold differences recorded for OPG. n = 3 technical replicate wells from a single donor.

**Figure 5:**
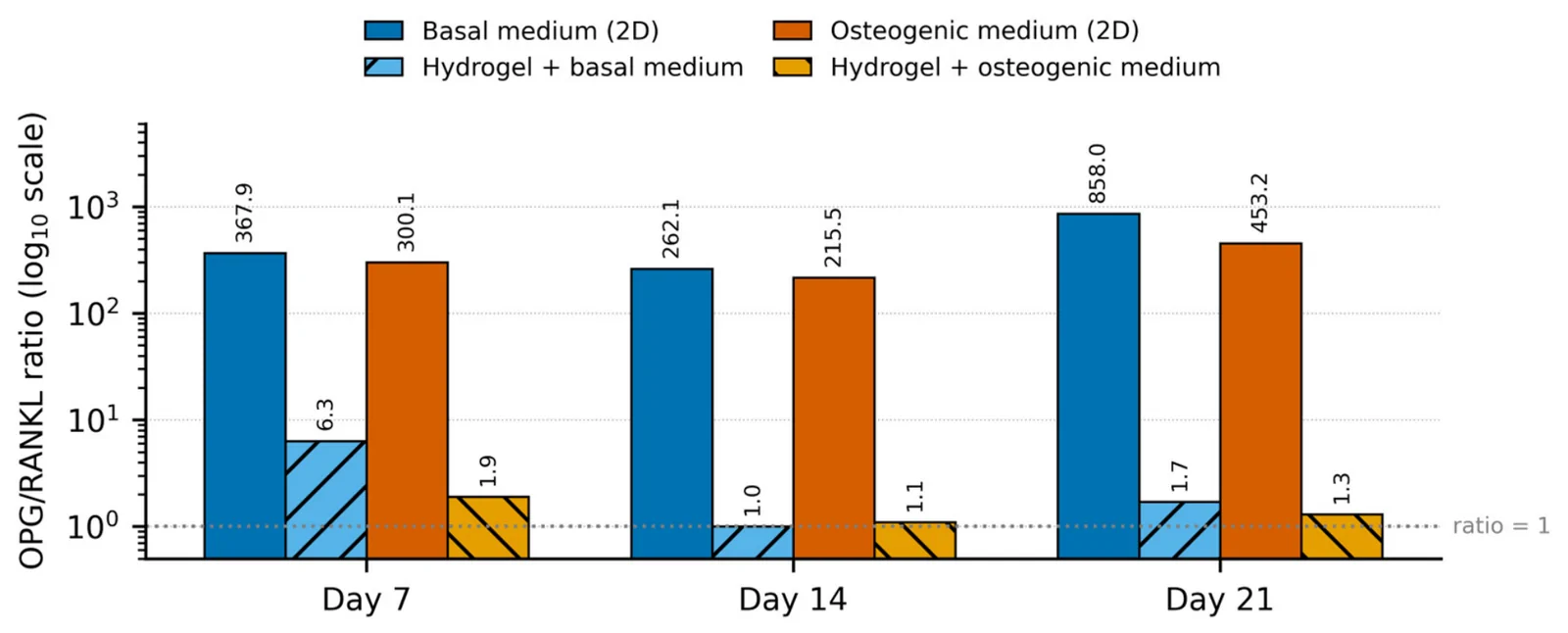
OPG/RANKL ratio at days 7, 14, and 21 for all experimental conditions. Hydrogel encapsulation dramatically reduced the ratio from 215–858 to 1.0–6.3, indicating selective retention of OPG by the matrix rather than increased osteoclastogenic activity.

All hydrogel groups in Figure 3, Figure 4 and Figure 5 and Table 1 and Table 2 are free-standing discs of the ternary formulation specified in Section 4.1: 10% (*w*/*v*) sodium alginate blended with chitosan (degree of deacetylation > 95%) and ionotropically cross-linked in 10% (*w*/*v*) CaCl_2_, differing only in nominal CBD load. The conditions designated hydrogel + BM and hydrogel + OM correspond to the CBD-free control (0 mg/mL), isolating the contribution of the matrix from that of the drug. The full concentration series (0.1, 0.5, 1, 6 and 12 mg/mL) was assayed in parallel at every interval in both media; across the series, supernatant OPG remained within the suppressed range of the CBD-free control and RANKL within 9–11 pg/mL, with no monotonic concentration dependence in either analyte.

Supernatant OPG concentration remained higher in the base medium than in osteogenic medium at all three time points (Figure 3; Table 1). This aligns with the pharmacological effects of the osteogenic cocktail, in which dexamethasone influences OPG expression in a time- and donor-dependent manner as differentiation advances [37]. Conventional cultures also exhibited substantial well-to-well variability, with coefficients of variation as high as 41.9% (Table 1). As all wells derive from a single donor, this dispersion is technical rather than inter-individual and does not support inference regarding donor-dependent behavior. This variability contrasts with the more consistent results observed in the hydrogel-exposed groups and explains the fluctuations in absolute OPG levels across time points.

The apparent suppression of detectable OPG in hydrogel supernatants is hypothesised to result from electrostatic adsorption within the alginate–chitosan polyelectrolyte network. This attribution rests on convergent indirect evidence and on the physicochemical contrast between the two measured proteins; it has not been demonstrated directly in this study, and the experiments required for confirmation are specified below. OPG is a 55 kDa glycoprotein with an isoelectric point (pI) of 8.5–9.0, carrying a net positive charge at physiological pH. This cationic character promotes ionic interaction with the negatively charged carboxylate groups (COO^−^) of alginate, analogous to the electrostatic adsorption documented for BMP-2 (pI ≈ 8.5) on oxidised alginate–carboxymethyl chitosan microspheres [18]. Polysaccharide polyelectrolyte multilayers retain adsorbed growth factors such as FGF-2 in bioactive form, yielding 1.8-fold higher mesenchymal stem cell densities than soluble delivery [38]. Chitosan–heparin multilayers similarly enable prolonged retention of FGF-2, confirming that chitosan-based polycation/polyanion systems function as effective protein reservoirs [39]. Additionally, scaffolds containing alginate and chitosan influence the availability of PDGF-BB and FGF-BB to DPSCs, indicating that these matrices modify the local paracrine microenvironment [40].

The hydrogel also improved the uniformity of the three-dimensional niche. Coefficients of variation (CV) for OPG production were significantly lower in hydrogel-exposed groups—2.20% and 5.41% at day 7; 5.99% and 14.32% at day 14—compared to conventional cultures, which showed 15.63% and 29.86% at day 7 and 41.87% and 13.10% at day 14. This suggests that the matrix offers a more consistent microenvironment, reducing cell-to-cell variability in secretion. These findings are consistent with reports indicating that alginate–gelatin hydrogel stiffness influences DPSC attachment, morphology, proliferation, and osteogenic differentiation in 3D cultures [24], and that peptide-functionalized GelMA hydrogels used in three-dimensional DPSC cultures enhance proliferation and osteogenic differentiation relative to two-dimensional monolayers [25].

A decrease in cell number, viability, or metabolic activity on the hydrogel is the main alternative explanation for the reduction in supernatant OPG. However, no viability, proliferation, or DNA-based assay was performed in this study, and this limitation is acknowledged. Nevertheless, three points argue against a general cell deficit as the sole cause. First, RANKL levels were measured in the same supernatants, from the same cells and wells: if 98–99.8% of secretory ability was lost, RANKL should have decreased proportionally, yet it remained at 9–11 pg/mL and was statistically similar to monolayer cultures at all time points. A non-specific cell loss would have reduced both analytes similarly; instead, a divergence of two to three orders of magnitude was observed. Second, cells recovered from hydrogel wells stained positively with Alizarin Red S on the underlying polystyrene at 7, 14, and 21 days, indicating a viable, osteogenically capable population remained throughout the culture. Third, these DPSCs, seeded on scaffolds of similar composition (chitosan/gelatin/alginate), maintained adhesion and viability [40], and matrices with similar stiffness like alginate/gelatin and GelMA support DPSC attachment and differentiation [24,25]. Note that the RANKL measurement is near the lower detection limit (5.0 pg/mL), so it is semi-quantitative rather than fully quantitative. To resolve this, live/dead assays, DNA quantification for normalizing analyte levels, and confocal imaging of cell distribution are needed.

Unlike the significant suppression of OPG, RANKL production by DPSCs stayed notably stable across all experimental conditions and time points, consistently ranging from 9 to 11 pg/mL (Figure 4). On day 7, RANKL levels measured were 9.67 ± 1.03 pg/mL in the base medium, 11.00 pg/mL in the hydrogel control plus base medium, 9.33 ± 0.82 pg/mL in osteogenic medium, and 11.00 pg/mL in the hydrogel control plus osteogenic medium, with a total mean of 10.06 ± 1.66 pg/mL (CV = 16.5%). By day 14, the levels were 9.25 ± 0.99 pg/mL, 9.83 ± 1.26 pg/mL, 9.00 pg/mL, and 9.33 ± 0.58 pg/mL, respectively. This trend continued at day 21, with values of 9.17 ± 0.41 pg/mL (base medium), 9.00 pg/mL (hydrogel + base medium), and 9.33 ± 0.58 pg/mL (hydrogel + osteogenic medium); the only exception was the osteogenic 2D group, which rose to 13.67 ± 0.52 pg/mL. Although this elevation reached statistical significance, its biological relevance is negligible given the 100–1000-fold differences observed in OPG across conditions. Additionally, testing CBD-loaded hydrogels at concentrations from 0 to 12 mg/mL confirmed that RANKL secretion remained within the 9–11 pg/mL range across both media at all time points. This consistency is likely due to the distinct physicochemical properties of the two proteins: RANKL is a smaller, membrane-associated cytokine (33 kDa as a trimer) with a pI of about 5.0–6.0, giving it a net negative charge at physiological pH. This charge likely causes electrostatic repulsion with the anionic alginate matrix, preventing adsorption and allowing free diffusion into the supernatant. Such differential retention significantly affects the OPG/RANKL ratio (Figure 4).

These results are consistent with DPSCs’ well-known secretory profile, characterized by high OPG and low RANKL levels, which confers a strong anti-osteoclastogenic effect. The study demonstrates that DPSCs in both media continuously release high levels of OPG with minimal RANKL secretion. This secretory pattern is well established for DPSCs; what is new here is that the alginate/chitosan matrix selectively binds OPG, altering the local OPG/RANKL balance, as explained below. Notably, contact with the hydrogel resulted in a significant (~98%) reduction in detectable supernatant OPG levels, regardless of cannabidiol concentration, including in the control hydrogel without CBD.

In samples without hydrogel, the OPG/RANKL ratio remained consistently high: 367.9 (BM) and 300.1 (OM) at day 7; 262.1 (BM) and 215.5 (OM) at day 14; and 858.0 (BM) and 453.2 (OM) at day 21, indicating a strong anti-osteoclastogenic phenotype of DPSCs. In contrast, hydrogel-exposed cultures displayed ratios close to one: 6.3 (BM) and 1.9 (OM) at day 7; 1.0 (BM) and 1.1 (OM) at day 14; and 1.7 (BM) and 1.3 (OM) on day 21. This suggests that the change does not reflect increased osteoclastogenic potential but rather indicates that OPG was sequestered within the hydrogel matrix, with RANKL remaining diffusible (Figure 5). Notably, matrix-retained OPG could create a localized anti-osteoclastogenic environment more effective than systemic soluble delivery. This is consistent with previous reports showing that sustained OPG release from hydrogels inhibits osteoclastogenesis and accelerates bone repair more effectively than a single bolus dose [41].

Two features make this electrostatic retention mechanistically plausible and address the concern that alginate and chitosan would neutralize each other. First, charge neutralization in an alginate/chitosan polyelectrolyte complex is incomplete when alginate is in excess, as in the present formulation (10% *w*/*v* alginate, additionally cross-linked with Ca^2+^): under these conditions the network keeps a net negative surface charge and abundant free carboxylate groups. This is consistent with the persistent asymmetric and symmetric –COO^−^ bands (≈1593 and 1411 cm^−1^) recorded by FTIR for this same hydrogel during its precursor characterization [42], which indicate that many carboxylates remain in the salt/Ca^2+^-bound form rather than being paired with chitosan amines. Second, OPG is intrinsically predisposed to bind anionic polysaccharides: through eight basic residues in its C-terminal heparin-binding domain, it binds heparin and heparan sulfate with nanomolar affinity, and this interaction is required for OPG retention and antiresorptive function on the osteoblast surface in vivo [43,44,45]. The alginate matrix can therefore act as a glycosaminoglycan-mimetic reservoir that captures OPG through the same physiological interface, whereas RANKL—net-negative at pH 7.4—is electrostatically excluded and remains diffusible. The differential behavior we observed (≈99% loss of supernatant OPG with essentially unchanged RANKL) is the expected signature of charge-selective retention.

Charge-based partitioning of the required magnitude is well documented at physiological ionic strength: in fibrillar hydrogels of defined electropotential, proteins are retained or released according to the sign of their net charge relative to the network, oppositely charged species releasing less than 10% of the initial load over one month while like-charged species diffuse essentially unhindered [46,47]. The retention inferred here (98–99.8%) is of comparable order, and the isoelectric points of OPG (≈8.5–9.0) and RANKL (≈5.0–6.0) span precisely the charge contrast that such networks discriminate. Charge-selective retention is therefore advanced as a hypothesis consistent with converging indirect evidence, not as an established result.

Cannabidiol did not produce a detectable effect on supernatant OPG or RANKL at any concentration tested (0.1–12 mg/mL). This null finding does not imply biological inactivity but rather reflects the dominance of the matrix-mediated retention over any drug-specific modulation measurable in the supernatant. In three-dimensional culture systems, the interconnected porous architecture of the alginate–chitosan matrix (pore sizes ≈10–46 µm [42]) combined with its ionic character creates a microenvironment where protein partitioning is governed by charge rather than by the presence of an exogenous bioactive agent. Furthermore, three-dimensional matrices induce polarised protein secretion toward the extracellular matrix rather than into the bulk medium [48], representing an inherent limitation of supernatant-based assays in 3D culture.

The pro-osteogenic effects of CBD on DPSCs are well documented: 2.5 μM CBD rescues TNF-α-inhibited proliferation, migration, and osteogenic differentiation [22], and it alleviates LPS-induced suppression of odonto/osteogenic differentiation in vitro [23]. In the present system, positive Alizarin Red staining in all hydrogel-exposed groups indicates that osteogenic differentiation occurred regardless of CBD load; however, the supernatant assay cannot discriminate CBD-specific effects when OPG is already sequestered by the matrix. Detection of CBD effects on intracellular osteogenic markers (ALP, Runx2, osteocalcin) and on matrix-associated OPG would require gene expression or immunocytochemistry assays not performed in this study. CBD is therefore retained in the formulation as a biologically motivated component whose effects require dedicated intracellular and release-kinetics characterisation in future work.

The physicochemical properties of this formulation were characterised in detail in a preceding study using the identical extrusion-printing protocol [42]; the parameters relevant to the present interpretation are summarised here. Pore dimensions (cryo-SEM: 12.81 ± 4.80 µm control, 16.39 ± 6.52 µm at 12 mg/mL; porosity 12.5–19.1%) exceed the hydrodynamic size of OPG (55 kDa) and RANKL (33 kDa) by approximately three orders of magnitude, ruling out steric sieving as an explanation for their differential recovery. The hydrogel exhibited equilibrium swelling of 1464 ± 28% at 72 h, water absorption > 90%, solid-like rheological behaviour (G′ > G″; yield stress 24.85–49.95 Pa), and compressive strength of 1261.8–3707.3 N, all modulated dose-dependently by CBD. FTIR confirmed retention of carboxylate bands (~1593 and 1411 cm^−1^), indicating that a substantial fraction of alginate carboxylates remains in the anionic salt form rather than paired with chitosan ammonium groups [42]—consistent with a net negative network charge capable of discriminating proteins by isoelectric point.

Several limitations qualify the conclusions drawn above. All data derives from a single DPSC donor (passage 6, technical triplicates), so replication across independent donors is the immediate next step [49]. Osteogenic differentiation was assessed qualitatively by Alizarin Red without cell-free controls or ALP quantification; the RANKL internal reference supports cell persistence but does not replace direct viability measurement. The electrostatic retention mechanism is supported by converging indirect evidence—pore size exclusion of steric effects, charge-contrast between OPG and RANKL, and the literature precedent in model polyelectrolyte systems—but awaits confirmation through adsorption isotherms, mass-balance quantification, and ζ-potential measurement. CBD release kinetics and matrix degradation under the culture conditions used remain to be characterized. Despite these constraints, the magnitude and selectivity of the observed OPG/RANKL dissociation identify the charge architecture of polysaccharide scaffolds as an active variable warranting systematic investigation.

The principal contribution of this work is the identification of a reproducible and selective dissociation between OPG and RANKL in the supernatant of DPSC-laden alginate/chitosan constructs. While DPSCs, CBD-mediated osteoinduction, and alginate/chitosan hydrogels have each been studied individually, their combination within a single system reveals that the polyelectrolyte matrix itself—independently of CBD—alters the apparent paracrine output of the cells it hosts. If the electrostatic retention hypothesis is confirmed, this effect could be exploited as a design principle: localising endogenous anti-osteoclastogenic signals within a 3D-printable scaffold, rather than relying on exogenous OPG delivery.

DPSCs were selected because they represent an accessible, minimally invasive cell source with a well-characterised OPG-dominant secretory profile <w:r><w:rPr><w:color w:val=“ff0000” /></w:rPr><w:t xml:space=“preserve”> [34], making them an ideal model</w:t></w:r> for testing whether the hydrogel selectively affects one arm of the axis. The charge-based retention mechanism, if confirmed, would be expected to operate independently of cell source for any OPG-secreting mesenchymal population; comparative studies with bone-marrow-derived MSCs are warranted but were beyond the scope of this work.

## 3. Conclusions

The alginate/chitosan/CBD hydrogel supported osteogenic commitment of DPSCs and produced a selective, reproducible dissociation between OPG and RANKL in the culture supernatant: OPG declined by 98–99.8% (*p* < 0.001) while RANKL remained stable at 9–11 pg/mL across all time points. Since a generalized cellular deficit would depress both analytes proportionally, the most parsimonious interpretation is charge-selective retention of cationic OPG (pI ≈ 8.5–9.0) within the anionic network, with simultaneous exclusion of anionic RANKL (pI ≈ 5.0–6.0). This observation establishes that the collapse of the supernatant OPG/RANKL ratio in polyelectrolyte scaffolds reflects matrix-mediated redistribution rather than a pro-osteoclastogenic shift and identifies macromolecular charge architecture as a potential design principle for localizing anti-resorptive signals at the peri-cellular level. Confirmation through adsorption isotherms, mass-balance quantification, and replication across independent donors will define the translational scope of this finding.

## 4. Materials and Methods

### 4.1. Materials

Sodium alginate (A2033), chitosan (448877, deacetylation degree > 95%), and calcium chloride (1086436) were obtained from Sigma-Aldrich (Merck KGaA, Darmstadt, Germany). Cannabidiol—CBD (99% purity; Avicanna, Toronto, ON, Canada). Analytical-grade ethanol was used in this study. Hydrogels were fabricated according to a previously published protocol with CBD doses of 0, 0.1, 0.5, 1, 6, and 12 mg/mL (Garzon et al., 2025) [42]. DPSCs were donated by the Center for Dental Research at Pontificia Universidad Javeriana (CIO-PUJ).

The ink was prepared as reported previously [42]: a 10% (*w*/*v*) sodium alginate solution in deionised water was blended with chitosan and, where applicable, with the CBD suspension; 3 mL of the 10% alginate solution was combined with 2 mL of the cannabinoid suspension and 0.4 mL of 10% (*w*/*v*) calcium chloride to yield the printable ink, and free-standing discs were obtained by immersion in 10% CaCl_2_ for 30 min. All hydrogel groups share this composition and differ only in nominal CBD load (0, 0.1, 0.5, 1, 6 and 12 mg/mL). Full physicochemical characterisation of the formulation, comprising SEM and cryo-SEM morphometry, FTIR, oscillatory rheology, uniaxial compression, shear testing, swelling and water absorption, is reported in [42] and summarised in Section 2.

Given its poor aqueous solubility, CBD was not added directly to the culture medium; instead, it was dissolved initially in 99% ethanol. After evaporating the ethanol, the residue was sonicated and incorporated into the alginate/chitosan ink before ionic cross-linking with Ca^2+^, following the validated procedure in [42]. The nominal CBD concentrations (0–12 mg/mL) thus denote the amount loaded into the hydrogel, not in the medium. Free-standing, Ca^2+^-cross-linked hydrogel discs were placed in the wells, and DPSCs (10,000 cells per well) were seeded onto the pre-formed hydrogels to create a three-dimensional cell–matrix contact culture (rows 2 and 3). Parallel wells without hydrogel served as the two-dimensional condition (rows 1 and 4).

### 4.2. Samples

Healthy teeth are transported to the CIO-PUJ laboratory in a contamination-free medium. Inside the laminar flow hood, they are carefully segmented with sterile instruments to extract the pulp tissue. This tissue is then cut into 1–2 mm^3^ fragments and cultured in 25 cm^2^ flasks using the explant method until cells migrate completely from each explant. From the primary culture, subcultures are carried out up to passage P6 and used in this study. As the subcultures develop, their cellular characteristics are monitored, and the presence of specific features determines their suitability for the experimental protocols, including those used in this research.

**Figure 6:**
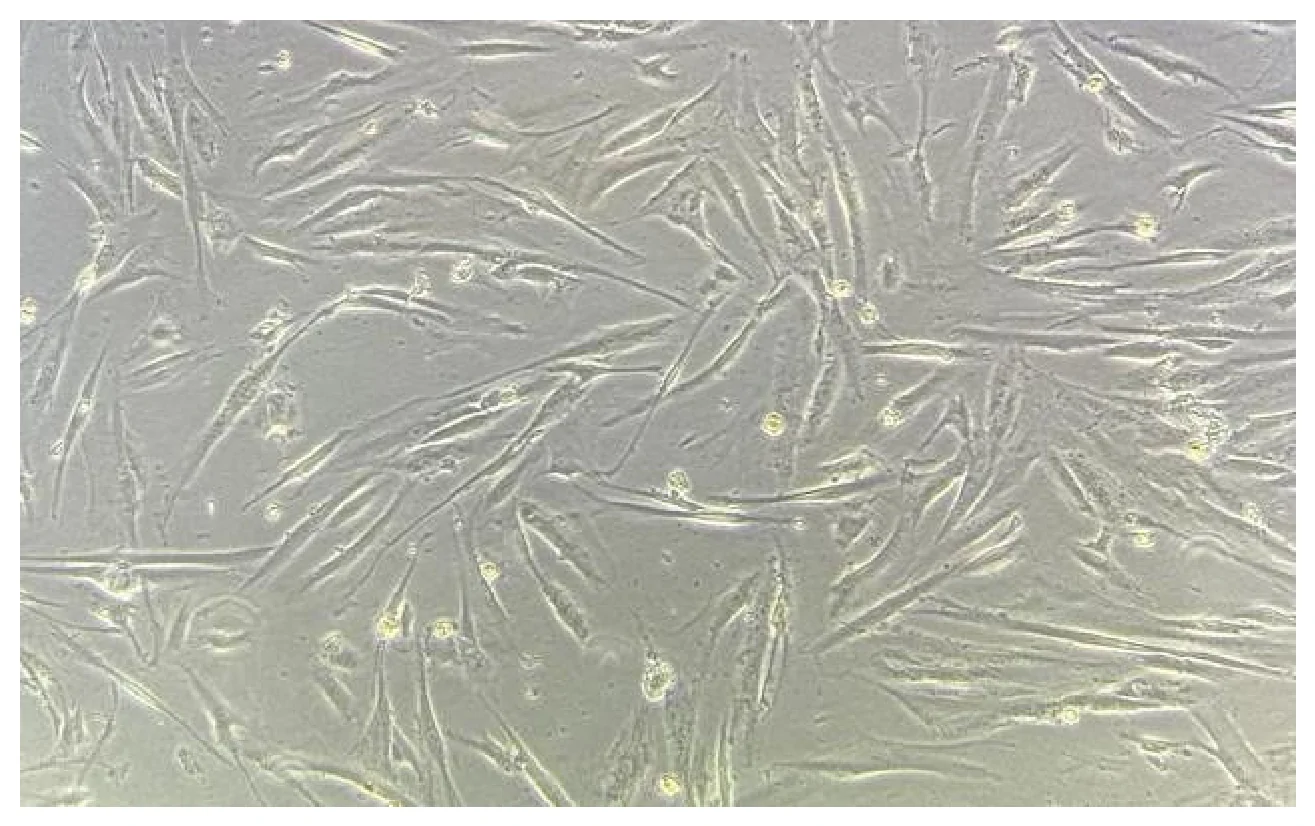
Stem cells derived from dental pulp DPSC. 10× microphotograph of a semi-confluent two-dimensional culture of human dental pulp-derived stem cells at P6.

Subculture cells at passage 6 (P6) were selected based on adherence to culture flasks, active migration in seeded explants, high-dilution seeding clonogenic capacity at P2, sustained proliferation with a doubling time of 1–1.5 days, positive MSC marker expression (CD90, CD29, CD146, CD73), negative lineage markers (CD45, CD324), and confirmed trilineage differentiation potential. Prior to the experiments, the mesenchymal identity of the DPSCs was confirmed by immunophenotyping and trilineage differentiation assays. The cells were plastic-adherent and displayed a mesenchymal surface-marker profile (positive for CD90, CD73, CD29, and CD146; negative for the hematopoietic/epithelial markers CD45 and CD324), together with osteogenic, adipogenic, and chondrogenic differentiation capacity, thereby fulfilling the minimal criteria for mesenchymal stromal cells. This same characterized DPSC population was subsequently used in a previous scaffold study [40] (Figure 6). Luminex cytokine quantification was performed on conditioned supernatants collected from triplicate wells per condition; all wells derived from a single biological donor at passage 6.

All experiments were performed with this single characterised DPSC population, obtained from one healthy 19-year-old male donor and used at passage 6. Each condition comprised triplicate wells, constituting technical replicates within one biological donor.

### 4.3. Culture Media

The culture medium consisted of α-MEM (Gibco, 12000-022, Waltham, MA, USA), supplemented with 20% fetal bovine serum (Gibco, 12657-029). It also contained 1% of a commercial antibiotic and antifungal solution (penicillin, streptomycin, and amphotericin B) (Gibco, 11580486), 1% Glutamax I (Gibco, 35050079), and 0.22% (2.2 g/L) sodium bicarbonate (MERCK, 137013). Cells were cultured in 25 cm^2^ flasks (Corning, 4761, New York, NY, USA) in a two-dimensional setup, maintained at 37 °C in an incubator with an environment of 95% air and 5% CO_2_.

To promote osteogenic differentiation, cells were cultured in an osteogenic medium composed of complete α-MEM, supplemented with 10 nM dexamethasone (Sigma Aldrich D4902, Burlington, VT, USA), 50 µM ascorbic acid (Sigma Aldrich A4544), and 10 mM β-glycerophosphate (Sigma-Aldrich G9422). These cultures were maintained throughout each observation period, and calcium deposition was assessed by Alizarin Red histological staining.

To stain the cultures induced for osteogenic differentiation, they were first washed twice with phosphate-buffered saline (PBS), then fixed in 4% paraformaldehyde (PFA) for 15 min. Afterward, the samples were washed twice as much with PBS, and 70% ethanol was added to cover the cell monolayer. For staining, Alizarin Red dye was prepared in ultrapure water, and its pH was adjusted to 4.3 using 1M sodium hydroxide (NaOH) (Sigma-Aldrich). The ethanol was removed from the fixed cells, and 2 mL of the diluted Alizarin Red dye was added to each sample. These were incubated at room temperature for 30 min with gentle agitation. Finally, the cells were washed three times with ultrapure water for visualization under a light microscope and image capture for analysis.

A third culture medium was used, consisting of α-MEM (Gibco, 12000-022) with 1% antibiotics (penicillin, streptomycin), antifungal (amphotericin B) (Gibco, 11580486), 1% Glutamax I (Gibco, 35050079), and 0.22% (2.2 g/L) sodium bicarbonate (MERCK, 137013). This ‘starving medium’ lacks fetal bovine serum, which contains proteins, amino acids, vitamins, minerals, and growth factors that can mask secreted molecules, complicating detection of secreted factors by Luminex. Thus, serum is removed before supernatant collection. Media were changed every two days.

DPSCs at passage P6 were seeded at 10,000 cells per well in 24-well plates. The layout: row 1 (wells 1–6) and row 4 (wells 1–6) were controls with only cells; rows 2 and 3 had cells on hydrogels with increasing drug concentrations—well 1: hydrogel alone (0 mg/mL), well 2: 0.1 mg/mL, well 3: 0.5 mg/mL, well 4: 1 mg/mL, well 5: 6 mg/mL, and well 6: 12 mg/mL. This was repeated across three plates for evaluation at 7, 14, and 21 days. After seeding, plates were cultured in α-MEM, induced toward osteogenesis, then serum-starved for 48 h in serum-free medium. Incubation times are in Table 3 and Table 4.

After seeding DPSC in multi-well plates, cells were induced to undergo osteogenic differentiation and then placed in starving medium. After two days in serum-free medium, supernatants were collected, and cells were stained with Alizarin Red. Cells in rows 1 and 2 were maintained in culture and serum-free for supernatant collection. Additionally, some DPSC were kept with culture medium after seeding, then with starving medium for the last two days. After two days in serum-free medium, supernatants were collected, and cells were stained with Alizarin Red as a negative control for differentiation.

### 4.4. Collection of Acellular Derivatives

After the 7, 14, and 21-day observation periods, cultured plates served two purposes: collecting supernatant and evaluating cell differentiation via Alizarin Red staining. Cells were cultured for 48 h in bovine fetal serum-free medium on established DPSC cultures. In rows 1 and 2, cells were cultured without osteogenic induction; row 1 had 2D proliferation, and row 2 had 3D growth in a hydrogel seeded with CBD at 0, 0.1, 0.5, 1, 6, and 12 mg/mL. The cell supernatants, containing acellular derivatives, were analyzed for OPG and RANKL concentrations with and without osteogenic induction by Luminex.

In rows 3 and 4, after initial culture, the medium was replaced with osteogenic differentiation medium. Row 3 had DPSC in 3D, while row 4 had cells in 2D. The 3D form in row 3 and 2 resulted from seeding cells in a hydrogel with varying CBD concentrations: 0, 0.1, 0.5, 1, 6, and 12 mg/mL.

The supernatants collected at each specified time and condition were lyophilized and stored frozen until analysis. On the day of testing, they were analyzed using Luminex to quantify analytes like OPG and RANKL, which are biomarkers released during the osteogenic differentiation of DPSCs across all conditions.

### 4.5. Determination of OPG and RANKL Concentration

OPG and RANKL were quantified by a magnetic bead-based multiplex immunoassay (Luminex xMAP technology; MILLIPLEX^®^ MAP, Merck-Millipore). OPG was measured with the Human Bone Magnetic Bead Panel (cat. no. HBNMAG-51K) and RANKL with the Human RANKL Magnetic Bead Single-Plex assay (cat. no. HRNKLMAG-51K-01), following the manufacturer’s instructions. Beads were read on a Luminex^®^ MAGPIX^®^ system with xPONENT^®^ software (version 4.2), and concentrations were interpolated from a five-parameter logistic standard curve. The minimum detectable concentrations reported by the manufacturer for these assays were 1.9 pg/mL for OPG and 5.0 pg/mL for RANKL; all reported values fell above these limits.

The lyophilised supernatants were first reconstituted with assay buffer to a final volume of 500 µL and coded to keep the analysis double-blind. Before starting the assay, all reagents were brought to room temperature (20–25 °C), and the manufacturer’s protocol was carefully reviewed. Standards, controls, and samples were arranged accordingly on a vertical plate map. Briefly, 200 µL of assay buffer was added to each well, then the plate was sealed and shaken for 10 min at room temperature; after this, the buffer was discarded, and residual liquid was removed by inverting and gently blotting the plate on absorbent paper. Next, 25 µL of standards or controls were added to designated wells, with assay buffer serving as the zero standard (background). For culture supernatant samples, cell culture medium was used as the matrix, and 25 µL of this matrix solution was added. Fifty microliters of each sample were then added to the corresponding wells. The bead mixture was vortexed to ensure even distribution, and 25 µL of the microspheres were dispensed into each well, with the vial vortexed intermittently to prevent sedimentation. The plate was sealed, covered with aluminum foil, and incubated with shaking for 16–18 h at 4 °C.

After incubation, the well contents were gently aspirated, and the plate was washed three times following the manufacturer’s washing procedure. Detection antibodies (50 µL) were added to each well, and the plate was sealed, protected from light, and incubated with shaking for 30 min (OPG) or 60 min (RANKL) at room temperature, without aspiration afterward. Then, 50 µL of Streptavidin-PE was added to each well, and the plate was sealed and incubated with shaking for 30 min at room temperature, protected from light. Following a final three-wash cycle, 100 µL of Sheath Fluid was added, and the microspheres were resuspended by shaking for 5 min. The plate was read using the Luminex^®^ MAGPIX^®^ system with xPONENT^®^ software, collecting a minimum of 50 microspheres per analyte. Results were reported as Median Fluorescence Intensity (MFI), and analyte concentrations were determined using a five-parameter logistic (5-PL) curve fit.

### 4.6. Statistical Analysis

Each experimental condition was evaluated in triplicate wells from a single biological donor (technical replicates); results are expressed as mean ± SD. Two-group comparisons used Student’s *t*-test and multi-group comparisons one-way ANOVA (significance threshold *p* < 0.05). Coefficients of variation are reported in Table 1. Because all replicates derive from one donor, the reported *p*-values quantify within-donor reproducibility and the study is framed as hypothesis-generating. The observed effect sizes (two to three orders of magnitude) and the invariance of RANKL as a within-plate internal reference support the robustness of the reported dissociation.

## Figures and Tables

**Table 1:** OPG concentration (pg/mL; mean ± SD) and coefficient of variation (CV = SD/mean × 100) by condition and time point, computed from the underlying triplicate measurements summarised in Figure 3. CVs were markedly lower in hydrogel-exposed groups, indicating a more uniform 3D microenvironment. All values derive from triplicate wells of a single biological donor and therefore represent technical, not biological, replicates.

Condition (Medium)	Day 7 Mean ± SD (CV%)	Day 14 Mean ± SD (CV%)	Day 21 Mean ± SD (CV%)
Basal, conventional culture	3558.0 ± 556.2 (15.6)	2424.2 ± 1014.9 (41.9)	7867.5 ± 676.8 (8.6)
Hydrogel + basal	69.3 ± 1.5 (2.2)	9.7 ± 0.6 (6.0)	15.7 ± 1.5 (9.6)
Osteogenic, conventional culture	2799.8 ± 836.0 (29.9)	1939.2 ± 746.6 (38.5)	6195.0 ± 811.9 (13.1)
Hydrogel + osteogenic	21.3 ± 1.2 (5.4)	10.7 ± 1.5 (14.3)	11.7 ± 2.1 (17.9)

**Table 2:** OPG/RANKL ratio by condition and time point.

Condition (Medium)	Day 7	Day 14	Day 21
Basal, conventional culture	367.9	262.1	858.0
Hydrogel + basal	6.3	1.0	1.7
Osteogenic, conventional culture	300.1	215.5	453.2
Hydrogel + osteogenic	1.9	1.1	1.3

**Table 3:** Culture maintenance time in each culture medium.

Observation Period	Culture Medium	Osteogenic Medium	Starving Medium
7 days	3 d	2 d	2 d
14 days	3 d	9 d	2 d
21 days	3 d	18 d	2 d

**Table 4:** Cell culture maintenance time as a negative control in each culture medium.

Observation Period	Culture Medium	Osteogenic Medium	Starving Medium
7 days	5 d	0 d	2 d
14 days	12 d	0 d	2 d
21 days	19 d	0 d	2 d

## Data Availability

The original contributions presented in this study are included in the article. Further inquiries can be directed to the corresponding author.
